# Impact of anti-VEGF treatment on development of proliferative diabetic retinopathy in routine clinical practice

**DOI:** 10.1186/s12886-024-03491-w

**Published:** 2024-05-31

**Authors:** Andrew A. Moshfeghi, Rahul N. Khurana, Hadi Moini, Steven Sherman, Kimberly Reed, Nick Boucher, Ehsan Rahimy

**Affiliations:** 1 Roski Eye Institute, Department of Ophthalmology, Keck School of Medicine, University of Southern California, Los Angeles, CA USA; 2 Northern California Retina Vitreous Associates, Mountain View, CA USA; 3 Regeneron Pharmaceuticals, Inc., Tarrytown, NY USA; 4 Vestrum Health, Naperville, IL USA; 5 Palo Alto Medical Foundation, 795 El Camino Real, Palo Alto, CA 94301 USA

**Keywords:** Anti–vascular endothelial growth factor, Diabetic macular edema, Disease progression, Non-proliferative diabetic retinopathy, Proliferative diabetic retinopathy

## Abstract

**Background:**

This study evaluated impact of anti–vascular endothelial growth factor (VEGF) treatment on proliferative diabetic retinopathy (PDR) development among patients with non-proliferative diabetic retinopathy (NPDR) in US real-world clinical practice.

**Methods:**

This was a retrospective analysis of electronic medical records (Vestrum Health; January 2013 to June 2019) of eyes with baseline NPDR, without DME, and naïve to anti-VEGF treatment at index DR diagnosis. Eyes that received anti-VEGF and/or laser treatment over the course of study before development of PDR constituted the treated cohort while the remaining including those treated with laser constituted the anti-VEGF naïve cohort. Survival analysis via Kaplan–Meier method evaluated time to DME and PDR development by baseline NPDR severity, with anti-VEGF treatment as censoring variable. Baseline factors affecting PDR development were analyzed using Cox multivariable regression, censoring for anti-VEGF treatment.

**Results:**

Among anti-VEGF–naive eyes, cumulative incidence of DME in eyes with mild (*n* = 70,050), moderate (*n* = 39,116), and severe NPDR (*n* = 10,692) at baseline was 27.1%, 51.2%, and 60.6%. Multivariable regression analysis identified baseline NPDR severity as the most significant predictor of PDR development over 48 months (hazard ratio [HR] [95% confidence interval {CI}] of 2.69 (2.65–2.72) for moderate vs mild NPDR and 6.51 (6.47–6.55) for severe vs mild NPDR). Cumulative incidence (95% CI) of PDR was 7.9% (7.4%–8.3%), 20.9%, (20.0%–21.7%) and 46.8% (44.4%–49.2%) over 48 months in eyes with mild, moderate, and severe NPDR at baseline, respectively. Among treated eyes with baseline severe NPDR, cumulative incidence of PDR at 48 months was 50.1% in eyes treated with laser (*n* = 546; HR [95% CI] vs no treatment: 0.8 [0.7–1.0]), 27.4% in eyes treated with anti-VEGF (*n* = 923; HR [95% CI]: 0.4 [0.4–0.5]), and 25.6% in eyes treated with anti-VEGF plus laser (*n* = 293; HR [95% CI]: 0.5 [0.4–0.7]) compared with 49.9% in eyes with no treatment (*n* = 8930).

**Conclusions:**

DME and PDR development rates increased with increasing baseline NPDR severity. Approximately half of anti-VEGF‒naive eyes with severe NPDR progressed to PDR within 4 years in US clinical practice. The progression rate from severe NPDR to PDR was approximately halved with anti-VEGF versus no treatment.

## Background

Diabetic retinopathy (DR) is the most common microvascular complication of diabetes [1] and the leading cause of vision impairment and blindness among working-age adults in the United States, primarily due to development of diabetic macular edema (DME) and proliferative DR (PDR) [1, 2]. Factors associated with an increased risk of DR include longer duration of diabetes and poorer glycemic control [3, 4].

DR is a progressive disease, and in clinical practice is classified as mild to moderate to severe non-proliferative DR (NPDR) to PDR [4]. According to one large electronic health records–based analysis, the risk of sustained blindness, defined as visual acuity (VA) readings of ≤ 20/200 at two separate visits ≥ 3 months apart that did not improve beyond 20/100, increased with the severity of DR [5]. Compared with mild NPDR cases, there was a 2.6 times increased risk of sustained blindness in patients with moderate NPDR after 2 years of DR diagnosis, and a 3.6 times increased risk among patients with severe NPDR [5].

Earlier studies reported that the risk of DME and PDR development increased with greater severity of NPDR [6–8]. However, even though management of DR and DME has improved over the last decade, only a limited number of studies have examined rates of these vision-threatening complications in the general DR population, particularly since the approval of anti–vascular endothelial growth factor (VEGF) therapies for treatment of DR and DME [9]. Although the impact of anti-VEGF therapy on the rate of DR progression has been demonstrated in clinical trials [10, 11], there is little evidence for this in the real-world from large databases.

The primary objective of this retrospective analysis was to evaluate the impact of anti-VEGF treatment on the development of PDR in eyes with NPDR in real-world clinical practice in the United States.

## Methods

### Data source

De-identified electronic medical records of patients with DR were obtained from the Vestrum Health Treatment and Outcomes database (Vestrum Health, Naperville, IL, USA). The Vestrum database comprised patient data obtained from 251 retina specialists at 54 private clinics from diverse geographic regions across the United States [12]. Data collected between January 1, 2013, and June 30, 2019, were extracted from the database using Structured Query Language queries. The records provided information about patient demographics, procedures, diagnoses, medications, and treatment outcomes. Patient electronic health records with pseudo-anonymized site and clinician data were de-identified in accordance with the Health Insurance Portability and Accountability Act prior to being made available for analysis in this study. The study was reviewed and considered exempt from institutional review board (IRB) approval by the WCG IRB (Seattle, Washington, USA), under 45 CFR § 46.104(d)(4).

NPDR, type 1 and type 2 diabetes, DME, vitreous hemorrhage, retinal detachment, retinal edema, and PDR were identified using International Classification of Diseases (ICD)-9 and ICD-10 codes and physician notes. Alternative definitions for PDR included the combination of codes for NPDR plus vitreous hemorrhage or retinal detachment.

### Study population

**Fig. 1:**
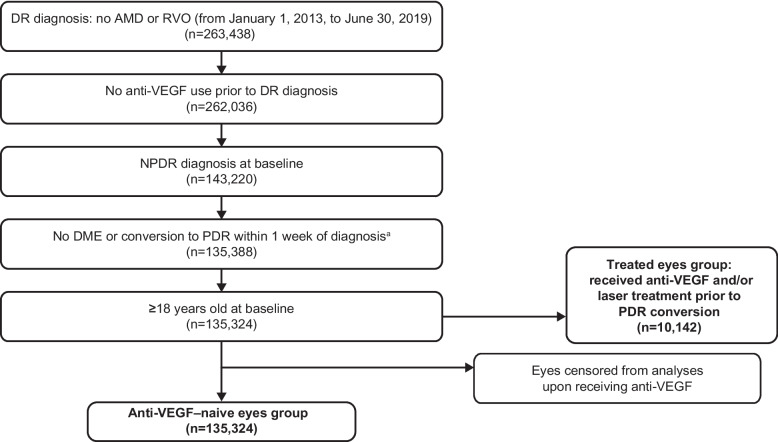
Study inclusion and exclusion criteria and eye flow diagram. ^a^Eyes with diagnosis codes for PDR or DME within 1 week of their NPDR diagnosis were excluded in addition to those with evidence of vitreous hemorrhage, retinal detachment, or retinal edema during the same period. AMD = age-related macular degeneration; DME = diabetic macular edema; DR = diabetic retinopathy; NPDR = non-proliferative diabetic retinopathy; PDR = proliferative diabetic retinopathy; RVO = retinal vein occlusion; VEGF = vascular endothelial growth factor

Eligible patients were ≥ 18 years of age with a diagnosis of NPDR without DME and were anti-VEGF naive at index DR diagnosis (defined using ICD-9 and ICD-10 diagnosis codes). Patients with age-related macular degeneration (AMD) or retinal vein occlusion (RVO) at index or during the study period, or those who developed DME or progressed to PDR within 1 week of index NPDR diagnosis, were excluded. This attrition resulted in a cohort of eyes with NPDR without DME that had not received anti-VEGF therapy prior to baseline (Fig. 1). In this cohort, eyes that received anti-VEGF therapy over the course of study before development of PDR were censored from the analyses, hence, constituting the anti-VEGF–naive cohort. Of note, eyes that received laser treatment during the study were included in this cohort. Eyes that received anti-VEGF therapy during the course of study before development of PDR were evaluated separately as the treated cohort.

In the treated cohort, eyes received anti-VEGF, laser, or both anti-VEGF and laser during the study before progressing to development of PDR. Anti-VEGF treatment refers to the use of ranibizumab, aflibercept, or bevacizumab. The type of laser treatment used was not available. Untreated eyes received no laser or anti-VEGF treatment prior to development of PDR.

### Outcomes

Cumulative incidences of DME and PDR development were assessed through 48 months by baseline NPDR severity (mild, moderate, or severe) in the anti-VEGF–naive eyes group (Fig. 1). Eyes that received anti-VEGF therapy over the course of study before development of PDR were censored from the analyses (eyes that received laser treatment during the study were included). Baseline factors affecting development of PDR were examined in the anti-VEGF–naive eyes group, censoring for anti-VEGF treatment. Cumulative incidence of PDR development by baseline NPDR severity was also assessed through 48 months in treated eyes by the treatment received during the study: no treatment, laser, anti-VEGF, or combination of anti-VEGF and laser.

### Statistical analysis

In the anti-VEGF–naive cohort, survival analysis by the Kaplan–Meier method was used to evaluate the cumulative incidence of DME by baseline NPDR severity, which was statistically compared using a log-rank test. Baseline factors associated with development of PDR in anti-VEGF–naive eyes were determined by multivariate analysis, and included age (per 5-year increments from 45–85 years), sex (male vs female), diabetes type (type 1 vs type 2), hypertension (yes vs no), presence of cataracts (yes vs no), VA (per 10-letter increments), intraocular pressure (≤ 21 mm Hg vs > 21 mm Hg), and NPDR severity (moderate vs mild, and severe vs mild). Hazard ratios (HRs) with 95% confidence intervals (CIs) for development of PDR were determined using a Cox multivariable regression model for adjusted analyses.

As a sensitivity analysis, the rate of PDR development in anti-VEGF–naive eyes was evaluated after censoring eyes that developed DME from the analysis (i.e., data from eyes that developed DME were not included in the analysis from that time point on).

Development of PDR by diabetes type and NPDR stage was assessed using a survival analysis in the anti-VEGF–naive cohort, with HR and 95% CI determined using Cox multivariable regression model. Development of PDR by different treatment types, stratified by baseline NPDR severity, was assessed using a survival analysis, with HR and 95% CI determined using unadjusted Cox regression.

## Results

### Study population

A total of 263,438 eyes with a diagnosis of DR and no AMD or RVO were identified in the database and assessed further for eligibility (Fig. 1). After applying inclusion and exclusion criteria, 135,324 eyes remained eligible for the study and were included in the anti-VEGF–naive cohort. Of these, 10,142 eyes were treated with laser and/or anti-VEGF before development of PDR during the study and were included in the treated cohort.

### Baseline characteristics

Among the anti-VEGF–naive eyes, 51.8% had mild NPDR, 28.9% had moderate NPDR, 7.9% had severe NPDR, and 11.4% had NPDR of unspecified severity at baseline (Table 1). Type 2 diabetes was markedly more common than type 1 diabetes (range: 55–62% vs 20–27% across NPDR severity groups, respectively) and was similar between NPDR severity groups. For the subset of eyes with available best-corrected VA measurements, mean (standard deviation [SD]) baseline VA was 73 (13.6) letters, with no notable differences between NPDR severity groups. Other baseline characteristics were similar across the NPDR severity groups.

**Table 1:** Baseline characteristics of anti-VEGF–naive and treated eyes by NPDR severity^a,b^

	**Anti-VEGF–naive eyes**					**Treated eyes**				
	**Mild** **(*n* = 70,050)**	**Moderate** **(*n* = 39,116)**	**Severe** **(*n* = 10,692)**	**Unspecified** **(*n* = 15,466)**	**Total** **(*n* = 135,324)**	**Mild** **(*n* = 2788)**	**Moderate** **(*n* = 3991)**	**Severe** **(*n* = 1768)**	**Unspecified** **(*n* = 1595)**	**Total** **(*N* = 10,142)**
NPDR severity, % of total	51.8	28.9	7.9	11.4	100	27.5	39.4	17.4	15.7	100
Age, mean (SD), years	64 (12.7)	63 (12.4)	59 (12.8)	65 (12.4)	64 (12.7)	65 (11.6)	63 (10.8)	60 (11.3)	65 (10.6)	63 (11.2)
Female, n (%)	34,589 (49.4)	19,151 (49.0)	4853 (45.4)	7493 (48.4)	66,068 (48.8)	1360 (48.8)	1953 (51.1)	816 (53.8)	730 (54.2)	4927 (51.4)
Diabetes type, n (%)										
Type 1	14,269 (20.4)	9908 (25.3)	2917 (27.3)	3975 (25.7)	31,069 (23.0)	650 (23.3)	944 (23.7)	464 (26.2)	409 (25.6)	2467 (24.3)
Type 2	43,500 (62.1)	22,429 (57.3)	5847 (54.7)	9293 (60.1)	81,069 (59.9)	1820 (65.3)	2569 (64.4)	1087 (61.5)	983 (61.6)	6459 (63.7)
Unspecified	12,281 (17.5)	6779 (17.3)	1928 (18.0)	2198 (14.2)	23,186 (17.1)	318 (11.4)	478 (12.0)	217 (12.3)	203 (12.7)	1216 (12.0)
DME in follow-up, n (%)	5649 (8.1)	8062 (20.6)	2781 (26.0)	3032 (19.6)	19,524 (14.4)	2231 (80.0)	3526 (88.3)	1375 (77.8)	1406 (88.2)	8538 (84.2)
Hypertension, n (%)	46,563 (66.5)	25,162 (64.3)	6424 (60.1)	10,722 (69.3)	88,871 (65.7)	1911 (68.5)	2581 (64.7)	1088 (61.5)	1070 (67.1)	6650 (65.6)
Cataracts, n (%)	25,812 (36.9)	14,031 (35.9)	3948 (36.9)	3908 (25.3)	47,703 (35.3)	935 (33.5)	1312 (32.9)	602 (34.0)	410 (25.7)	3259 (32.1)
VA,^c^ mean (SD), letters	73 (13.2)	72 (13.8)	72 (14.2)	72 (14.0)	73 (13.6)	68 (22.2)	70 (17.4)	69 (19.1)	69 (19.1)	69 (19.3)
IOP,^d^ mean (SD), mm Hg	16 (3.8)	16 (3.9)	16 (4.0)	16 (3.7)	16 (3.7)	16 (4.1)	16 (4.3)	16 (4.2)	16 (3.8)	16 (4.1)

*DME* diabetic macular edema, *IOP* intraocular pressure, *NPDR* non-proliferative diabetic retinopathy, *SD* standard deviation, *VA* visual acuity, *VEGF* vascular endothelial growth factor

^a^Ethnicity data were not available

^b^Both eyes from a patient could be included if both eyes qualified

^c^Analysis included 107,777 untreated eyes (mild, *n* = 56,627; moderate, *n* = 30,883; severe, *n* = 8253; unspecified, *n* = 12,014) and 8047 treated patients (mild, *n* = 2272; moderate, *n* = 3166; severe, *n* = 1388; unspecified, *n* = 1221)

^d^Analysis included 132,308 untreated eyes (mild, *n* = 68,730; moderate, *n* = 38,193; severe, *n* = 10,468; unspecified, *n* = 14,917) and 9841 treated patients (mild, *n* = 2723; moderate, *n* = 3870; severe, *n* = 1723; unspecified, *n* = 1525)

Among treated eyes, 27.5% had mild NPDR, 39.4% had moderate NPDR, 17.4% had severe NPDR, and 15.7% had unspecified NPDR at baseline (Table 1). Patients who received treatment for severe NPDR at baseline were relatively younger, more often male, and more often with type 1 diabetes compared with treated eyes with mild or moderate baseline NPDR severity. For the subset of eyes with available best-corrected VA measurements, mean (SD) VA was 69 (19.3) letters, with no notable differences between NPDR severity groups. The incidence of DME during follow-up was 78–88% across baseline NPDR severity groups in treated eyes. Anti-VEGF therapy was the most frequently used treatment across all NPDR severity groups (52–62%), followed by laser alone (21–31%) and anti-VEGF plus laser (13–18%). Use of laser alone was most common in eyes with severe baseline NPDR (31%) compared with other NPDR severities.

### Cumulative incidence of DME by baseline NPDR severity in anti-VEGF–naive eyes

**Fig. 2:**
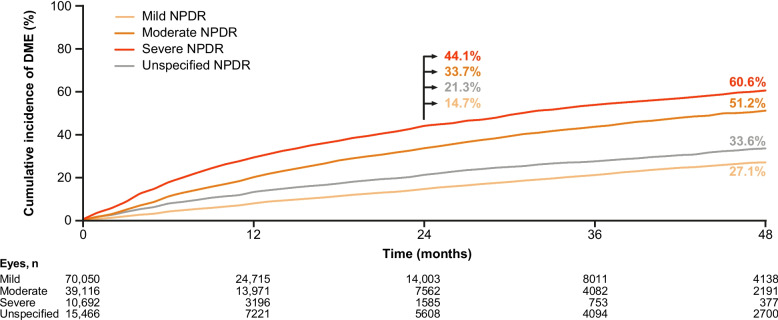
Cumulative incidence of DME by baseline NPDR severity in anti-VEGF–naive eyes. Anti-VEGF‒naive eyes included eyes that received laser treatment during the study. DME = diabetic macular edema; NPDR = non-proliferative diabetic retinopathy; VEGF = vascular endothelial growth factor

Cumulative incidence of DME in anti-VEGF–naive eyes over 48 months increased with the severity of NPDR at baseline (Fig. 2). Cumulative incidence of DME in eyes with mild, moderate, and severe NPDR at baseline was 14.7%, 33.7%, and 44.1% at 24 months, and 27.1%, 51.2%, and 60.6% at 48 months, respectively (Fig. 2).

### Baseline characteristics affecting development of PDR in anti-VEGF–naive eyes

**Fig. 3:**
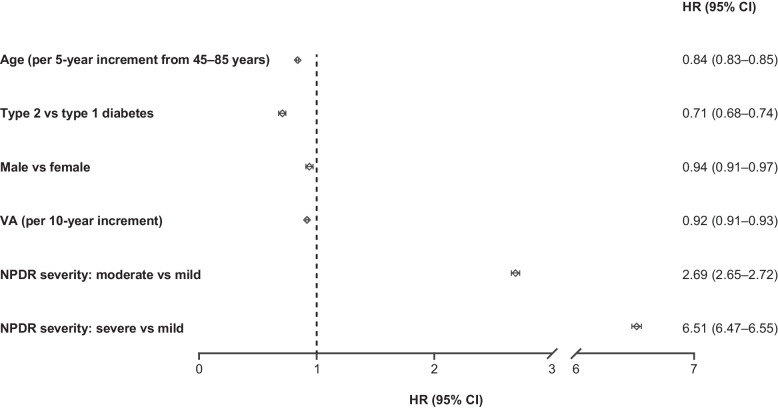
Baseline characteristics affecting development of PDR in anti-VEGF–naive eyes. This analysis included only eyes with vision of ≥ 20/1000 (ETDRS letters = 0). Anti-VEGF‒naive eyes included eyes that received laser treatment during the study. CI = confidence interval; ETDRS = Early Treatment Diabetic Retinopathy Study; HR = hazard ratio; NPDR = non-proliferative diabetic retinopathy; PDR = proliferative diabetic retinopathy; VA = visual acuity; VEGF = vascular endothelial growth factor

Baseline factors significantly affecting development of PDR over 48 months are shown in Fig. 3. Among them, diabetes type (type 1 vs type 2) and baseline NPDR severity were the factors most strongly associated with development of PDR. Eyes of patients with type 2 diabetes had a 29% lower risk of developing PDR compared with those with type 1 diabetes (HR: 0.71; 95% CI: 0.68–0.74), while an increased risk of developing PDR was observed in eyes with moderate NPDR (HR: 2.69; 95% CI: 2.65–2.72) and severe NPDR (HR: 6.51; 95% CI: 6.47–6.55), compared with eyes with mild NPDR (Fig. 3).

### Incidence of PDR by diabetes type and NPDR severity in anti-VEGF–naive eyes

Cumulative incidence of PDR with versus without censoring for DME was 8.4% versus 8.7% at 24 months and 14.9% versus 15.5% at 48 months, indicating DME had no significant impact on the rate of development of PDR. Therefore, eyes were excluded from analysis for the period following DME development.

**Fig. 4:**
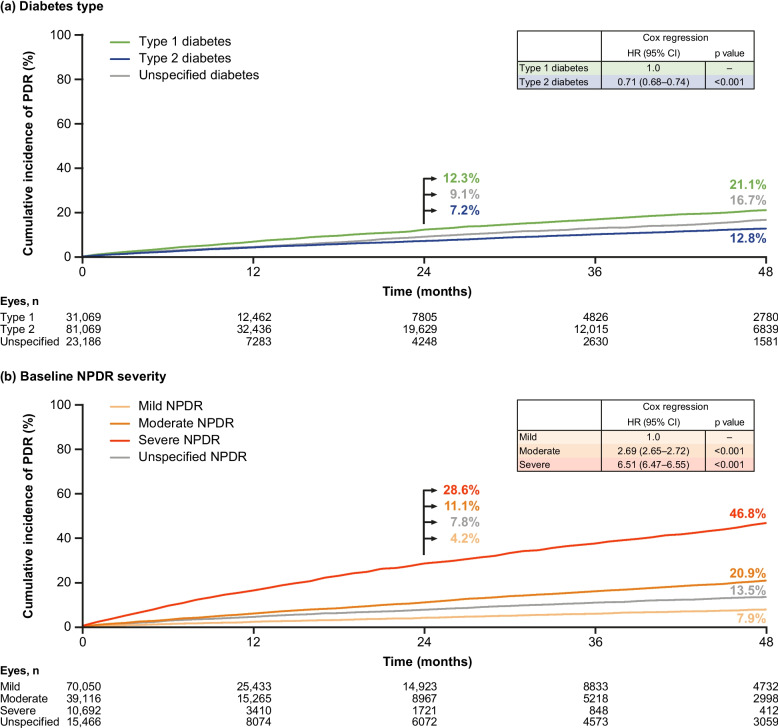
Cumulative incidence of progression from NPDR to PDR in anti-VEGF–naive eyes. Shown are cumulative incidence of progression from NPDR to PDR in anti-VEGF–naive eyes by (**a**) diabetes type and (**b**) baseline NPDR severity. Anti-VEGF‒naive eyes included eyes that received laser treatment during the study. CI = confidence interval; HR = hazard ratio; NPDR = non-proliferative diabetic retinopathy; PDR = proliferative diabetic retinopathy; VEGF = vascular endothelial growth factor

Cumulative incidence (95% CI) of PDR development in eyes of patients with type 2 versus type 1 diabetes was 7.2% (6.9%–7.5%) versus 12.3% (11.7%–12.8%) at 24 months, and 12.8% (12.4%–13.3%) versus 21.1% (20.2%–22.0%) at 48 months (*p* < 0.001 for both comparisons) (Fig. 4a). Cumulative incidence (95% CI) of PDR development in eyes with mild, moderate, and severe NPDR was 4.2% (3.9%–4.4%), 11.1% (10.6%–11.6%), and 28.6% (27.2%–30.1%) at 24 months, and 7.9% (7.4%–8.3%), 20.9% (20.0%–21.7%), and 46.8% (44.4%–49.2%) at 48 months, respectively (*p* < 0.001 for all comparisons) (Fig. 4b).

### Development of PDR in treated eyes

**Fig. 5:**
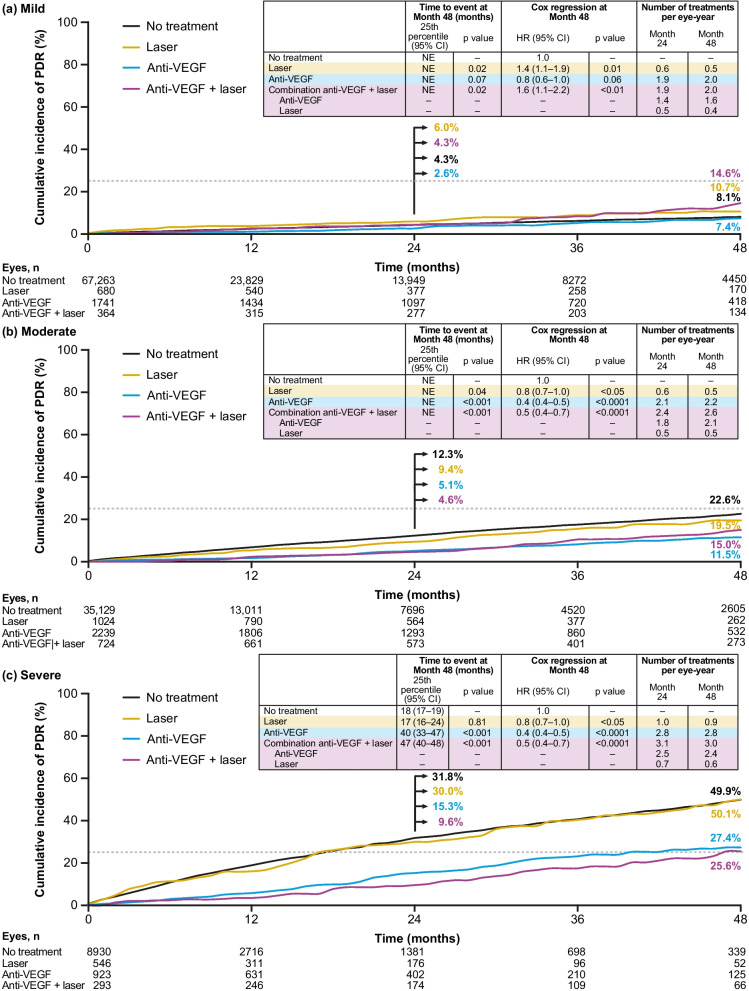
Cumulative incidence of PDR development by treatment during the study and baseline NPDR severity. Shown are cumulative incidences of PDR development by treatment during the study with baseline NPDR severity: **a** mild, (**b**) moderate, and (**c**) severe. The horizontal dotted line represents the 25th percentile. Treated eyes included eyes that received anti-VEGF, laser treatment, or anti-VEGF plus laser treatment prior to developing PDR. CI = confidence interval; HR = hazard ratio; NE = not evaluable; NPDR = non-proliferative diabetic retinopathy; PDR = proliferative diabetic retinopathy; VEGF = vascular endothelial growth factor

Given that the length of follow-up was variable, exposure was analyzed as number of treatments per eye-year of observation. Overall, number of treatments per eye-year over 24 and 48 months of follow-up were similar within each level of DR severity but trended higher across increasing levels of DR severity from mild to severe (Fig. 5 inset). Over 48 months of follow-up, the number of treatments per eye-year with laser, anti-VEGF, and combination of anti-VEGF and laser was, respectively, 0.5, 2.0, and 2.0 in eyes with mild NPDR, 0.5, 2.2, and 2.6 in eyes with moderate NPDR, and 0.9, 2.8, and 3.0 in eyes with severe NPDR.

Cumulative incidence of PDR development at Months 24 and 48 increased with greater NPDR severity more distinctly in the no-treatment and laser groups compared with the anti-VEGF group (Fig. 5). A lower cumulative incidence of PDR in eyes treated with anti-VEGF agents compared with those not treated or that received laser alone was evident at moderate NPDR severity and became more pronounced with severe NPDR, as seen by the increasing separation between the cumulative incidence curves in Fig. 5.

In eyes with severe NPDR, cumulative incidences of PDR at 24 months were 31.8% with no treatment, 30.0% with laser, 15.3% with anti-VEGF, and 9.6% with anti-VEGF plus laser. Corresponding incidences at 48 months were 49.9%, 50.1%, 27.4%, and 25.6%, respectively. At 48 months, HRs for development of PDR were 0.8 (95% CI: 0.7–1.0) with laser versus no treatment (*p* = 0.02), 0.4 (95% CI: 0.4–0.5) with anti-VEGF versus no treatment (*p* < 0.0001), and 0.5 (95% CI: 0.4–0.7) with anti-VEGF plus laser versus no treatment (*p* < 0.0001). Median time to development of PDR was also significantly longer with anti-VEGF treatment (40 months) and anti-VEGF plus laser (47 months) compared with no treatment (18 months; *p* < 0.001 for both comparisons). Median time to development of PDR with laser alone was 17 months, which was not statistically different versus no treatment (*p* > 0.05).

## Discussion

This retrospective study of eyes with NPDR found presenting disease severity as a key predictor of DME and PDR development among anti-VEGF–naive eyes. The incidence of PDR development was significantly lower in eyes treated with anti-VEGF agents compared with eyes that were untreated or received laser alone. These findings suggest that when left untreated, approximately 50% of eyes with severe NPDR progressed to PDR within 4 years, which is approximately twice the proportion of eyes treated with anti-VEGF progressing to PDR in routine clinical practice in the United States.

Few studies have evaluated the rate of PDR development in the general NPDR population in the United States. Earlier studies such as that conducted by the Early Treatment Diabetic Retinopathy Study (ETDRS) group reported that 79.5% of eyes with severe NPDR developed PDR over 5 years of follow-up from enrollment (1980–1985) [6]. This rate is higher than that found in the present study and likely reflects improvement in management of diabetes and DR since the time the ETDRS study was conducted. More recently, an analysis of retrospective claims data of treatment-naive US patients with mild or moderate NPDR reported 5-year progression rates to severe NPDR or PDR (5.8% for patients with mild NPDR, 17.6% for patients with moderate NPDR) that were similar to the 4-year PDR development rates reported in the present analysis (7.9% for patients with mild NPDR, 13.5% for patients with moderate NPDR) [9]. Several other studies have reported rates of PDR development, but for subpopulations of patients such as African Americans or only individuals with type 1 diabetes [13, 14]. Among previous studies that included both patients with type 1 and 2 diabetes, relative risk of PDR development was not examined by diabetes type [6, 9, 15]. Our study found that type 2 diabetes was associated with 29% lower risk of PDR development compared with type 1 diabetes. Older age was also associated with lower risk of PDR development, suggesting that PDR events were more associated with younger patients who presumably had a shorter duration of diabetes. However, the impact of duration of diabetes as well as markers of disease control such as glycosylated hemoglobin levels on the rate of PDR development could not be evaluated as data for these risk factors were not available. Regardless, these findings highlight the importance of diabetes type among other risk factors for development of PDR [15].

Another important finding of the current study is the impact of anti-VEGF therapy on development of PDR. Our results indicate that, in eyes with moderate or severe NPDR at baseline, treatment with anti-VEGF (with or without laser) resulted in a lower incidence of PDR development, with a longer time to PDR development, compared with eyes that received laser alone or no treatment. The number of anti-VEGF treatments per eye-year was ~ 2.5, which was lower than the number of scheduled injections in the first year of PANORAMA or Protocol W (approximately 6 injections with initial dosing followed by injections every 16 weeks) but was similar to a mean of 2.6 injections with a maintenance regimen of injections every 16 weeks in the second year of PANORAMA [10, 11]. This suggests that a few anti-VEGF injections per year may help improve the prognosis of these patients. In contrast, laser alone provided no benefit compared with no treatment in preventing development of PDR in patients with moderate or severe NPDR.

The finding of a reduced risk of PDR development in eyes with severe NPDR with proactive anti-VEGF treatment compared with no treatment is consistent with recent results from clinical trials that showed a similar extent of reduction in disease progression with anti-VEGF treatment in similar patient populations [10, 11, 16, 17]. Among patients with severe NPDR in this study, a small proportion were treated (17%), although the majority of those (69%) received treatment with an anti-VEGF agent (with or without laser). This low usage of anti-VEGF agents for treating severe NPDR in clinical practice likely reflects current DR treatment guidelines that support monitoring of patients with severe NPDR without DME [4, 18]. However, these results may have underestimated the benefit of anti-VEGF treatment versus no treatment, as the use of anti-VEGF agents was relatively lower in this study compared with PANORAMA and Protocol W [10, 11]. This low number of treatments likely reflects early adoption by clinicians using these treatments in select patients with NPDR perceived as having a high risk of progression. Further research is warranted to evaluate whether wider use of anti-VEGF agents among patients with NPDR yields greater benefit at the population level in routine clinical practice.

Strengths of this analysis include the large sample size drawn from clinical settings over an extended period of time (4-year follow-up) during a recent time period from 2013 to 2019. Such real-world evidence is likely to be reflective of the heterogeneity of a US NPDR patient population and current management of NPDR. Limitations include the retrospective nature of the study and use of electronic medical records as a data source. This restricted the ability to identify factors associated with DR progression, considering the limited availability of baseline patient demographics and eye characteristics, fluorescein angiography/fundus photography images, and socioeconomic information. Similarly, the type of laser used was not available. The Vestrum database was limited to data from 54 private clinics, unlike national registries and insurance data that have larger data sets [5, 19, 20]. This analysis excluded a subset of patients (1.9%) with AMD or RVO who were diagnosed with NPDR during the follow-up time, as potential management of AMD or RVO could have impacted interpretation of the results. It is unlikely that this exclusion impacted the conclusions given the small size of this subpopulation. There were no corrections for multiple comparisons. Reliance solely on ICD codes as the primary source for disease severity was a further limitation. The study was not designed to compare effects of different anti-VEGF agents, and the analysis of treatment effect was performed without accounting for the timing of the treatment during follow-up, the number of injections administered, or the stage of NPDR at the time of treatment. Furthermore, treatment patterns such as alternative loading dose periods and dosing schedules were not captured in the database, and the treating clinicians’ reasons for initiating treatment (laser or anti-VEGF) were not available. A possible source of bias may have arisen from clinicians choosing to treat patients deemed more likely to respond to treatment, particularly when a new treatment became available. The results of this study should be interpreted with caution and may not be used to establish a causal relationship between treatment with anti-VEGF therapy and reduced rates of PDR development.

## Conclusions

Among patients with NPDR in routine US clinical practice studied over a 4-year period, higher NPDR severity at baseline was associated with increased rates of PDR development, consistent with prior studies [6, 9]. In eyes with severe NPDR, development of PDR occurred in approximately half of patients in the absence of treatment. However, the rate of PDR development was substantially lower in eyes with severe NPDR that received anti-VEGF therapy compared with eyes that were untreated or received laser alone. Findings from this current analysis that reflects current trends may help inform management of patients with NPDR and highlight the potential benefits of using anti-VEGF therapies, particularly to treat eyes with severe NPDR, on an individualized basis.

## Data Availability

All available relevant data are included in the manuscript.

## Abbreviations

AMD: Age-related macular degeneration
CI: Confidence interval
DME: Diabetic macular edema
DR: Diabetic retinopathy
ETDRS: Early Treatment Diabetic Retinopathy Study
HR: Hazard ratio
ICD: International Classification of Diseases
IOP: Intraocular pressure
NE: Not evaluable
NPDR: Non-proliferative diabetic retinopathy
PDR: Proliferative diabetic retinopathy
RVO: Retinal vein occlusion
SD: Standard deviation
VA: Visual acuity
VEGF: Vascular endothelial growth factor
